# The Advanced BioTRIZ Method Based on LTE and MPV

**DOI:** 10.3390/biomimetics11010023

**Published:** 2026-01-01

**Authors:** Zhonghang Bai, Linyang Li, Yufan Hao, Xinxin Zhang

**Affiliations:** 1 National Engineering Research Center for Technological Innovation Method and Tool, Tianjin 300401, China; 202332305058@stu.hebut.edu.cn (L.L.);; 2 School of Architecture and Art Design, Hebei University of Technology, Tianjin 300132, China; zhangxinxin@hebut.edu.cn; 3 Key Laboratory of Healthy Human Settlements in Hebei Province, Tianjin 300132, China; 4 School of Mechanical Engineering, Hebei University of Technology, Tianjin 300401, China

**Keywords:** BioTRIZ, technological evolution, biologically inspired design

## Abstract

While BioTRIZ is widely employed in biomimetic design to facilitate creative ideation and standardize workflows, accurately formulating domain conflicts and assessing design schemes during critical stages—such as initial concept development and scheme evaluation—remains a significant challenge. To address these issues, this study proposes an advanced BioTRIZ method. Firstly, the theory of technological evolution is integrated into the domain conflict identification stage, resulting in the development of a prompt framework based on patent analysis to guide large language models (LLMs) in verifying the laws of technological evolution (LTE). Building on these insights, domain conflicts encountered throughout the design process are formulated, and inventive principles with heuristic value, alongside standardized biological knowledge, are derived to generate conceptual solutions. Subsequently, a main parameter of value (MPV) model is constructed through mining user review data, and the evaluation of conceptual designs is systematically performed via the integration of orthogonal design and the fuzzy analytic hierarchy process to identify the optimal combination of component solutions. The optimization case study of a floor scrubber, along with the corresponding experimental results, demonstrates the efficacy and advancement of the proposed method. This study aims to reduce the operational difficulty associated with implementing BioTRIZ in product development processes, while simultaneously enhancing its accuracy.

## 1. Introduction

Biologically inspired design represents a critical and fundamental strategy in the evolutionary advancement of products toward idealized states [1,2]. The integration of biological system knowledge into engineering applications through biomimetic design not only enhances the aesthetic appeal and emotional significance of products but also fosters innovation across multiple dimensions, including structure, function, and behavior [3,4,5]. It is essential to emphasize that the success of biomimetic strategies fundamentally relies on two critical factors: (1) the designer’s comprehensive understanding of the biological prototype, and (2) the evaluation of the biological prototype’s applicability within engineering contexts. To facilitate efficient and accurate biomimetic design, existing studies have introduced various methods, which are primarily categorized into function-oriented approaches [6,7] and domain knowledge-driven approaches [8,9]. However, in comparison to BioTRIZ, these methods often lack a systematic toolchain and an extensive knowledge base. Recently, BioTRIZ has achieved notable advancements in design domains such as mechatronic systems [10], sustainable design [11], and intelligent robotics [12]. By integrating biomimetic principles with the theory of inventive problem solving (TRIZ), BioTRIZ represents a systematic innovation methodology [13].

Specifically, BioTRIZ, based on analysing the similar functional implementation mechanisms between product systems and biological systems, guides designers to adopt reverse engineering thinking, promoting innovation and problem-solving by modifying specific elements and the relationships between them [14,15]. However, there are still three key challenges hindering the practice of BioTRIZ. (C1) Design objectives are vague at the front end of the design process. Designers often need to analyse user feedback to identify requirements and refine design goals [16,17], thereby effectively integrating relevant biological knowledge during function construction and problem-solving. However, the inherent ambiguity of natural language increases the risk of design objectives deviating from actual user needs [18]. (C2) The knowledge space of designer is limited compared to that of biological systems. The generation of design ideas is often constrained by designers’ attention within their subjectively familiar domains, leading to the neglect of existing or mature competing systems in other fields [19]. Although large language models (LLMs) have shown considerable potential in addressing this issue, they are prone to hallucinations and limited by prompt design. (C3) The abstract reasoning involved in the BioTRIZ implementation process complicates the design procedure. Existing studies commonly criticise the excessive reliance on designers’ experience when mapping design cases to BioTRIZ principles. Moreover, function-oriented development often neglects user needs, which are pertinent for the successful commercialization of biomimetic products. This limitation increases the uncertainty of achieving effective design solutions.

This study seeks to address the identified challenges (C1–C3) by enhancing the BioTRIZ methodology through the incorporation of the theory of technological evolution and the main parameter of value (MPV). The principal contributions of this research are as follows:

1. A robust framework is developed that leverages LLMs to analyze the evolutionary trajectories of products based on pertinent patent data (C2) and to forecast optimal design objectives (C1);
2. The integration of the laws of technological evolution (LTE) and ideal goals, as derived from TRIZ theory, addresses challenges associated with defining the operation domain of BioTRIZ (C3) and diminishes the complexity of biological knowledge;
3. The establishment of an evaluation system that combines MPV with biomimetic indicators through the inclusion of online product reviews, alongside the application of orthogonal design and fuzzy analytic hierarchy process (Fuzzy-AHP) for the effective quantitative assessment of integrated design schemes (C3).

The remainder of this paper is structured as follows: Section 2 presents a review of the pertinent literature; Section 3 offers a comprehensive introduction to the proposed framework and methodologies; Section 4 demonstrates the efficacy of the proposed methods through a case study focused on the optimization design of a floor cleaning machine; and Section 5 provides a summary and outlines potential directions for future research.

## 2. Related Works

### 2.1. BioTRIZ

**Figure 1:**
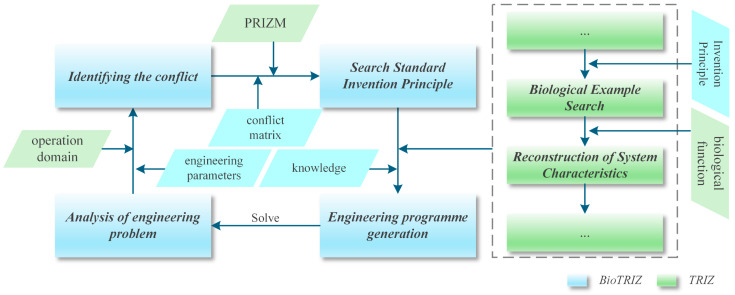
The TRIZ-based process modification and knowledge expansion of BioTRIZ.

The TRIZ theory asserts that the occurrence of technical problems can typically be attributed to the enhancement of one design parameter resulting in the degradation of another parameter [20,21]. Building on this premise, the conflict matrix is structured as a matrix comprising 39 engineering parameters along both its rows and columns, each representing design parameters exhibiting opposing tendencies. The application of this matrix involves four key steps, as illustrated within the blue section of Figure 1. Nevertheless, Vincent et al. [22] have noted that TRIZ was originally developed for technical and non-biological systems, and its direct application to biological contexts often results in the oversight of critical knowledge and structural considerations. Consequently, Vincent et al. validated and extended the conflict matrix through a comprehensive analysis of a diverse array of biological phenomena and functions. This effort culminated in the development of the BioTRIZ theoretical framework, which incorporates 500 biological phenomena and 270 functions [23]. The practical application of this framework can be delineated in four primary steps: (1) defining domain-specific conflict parameters grounded in engineering challenges and biological insights; (2) identifying relevant inventive principles and biological prototypes within the PRIZM matrix; (3) optimizing the engineering system by leveraging the functions and implementation mechanisms of the selected biological prototypes; and (4) proposing corresponding design solutions [24,25,26]. These enhancements are depicted in the green section of Figure 1. The PRIZM matrix is shown in Table 1, the numbers within the matrix represent the identification numbers of the TRIZ principles.

Salmaan et al. [27] employed BioTRIZ to develop a roof featuring an open cellular structure, effectively addressing the issue of low thermal mass associated with conventional insulation materials in cold climate conditions. Bogatyreva et al. [28] utilized BioTRIZ to resolve contradictions and obstacles encountered during the collaborative development of eco-innovations and corresponding business models. Bai et al. [29], drawing upon BioTRIZ, constructed a multi-biological prototype bionic design model aimed at identifying the optimal combination of biological prototypes. Zhou et al. [12] integrated relevant BioTRIZ theories to optimize feature transfer processes, thereby enabling a more comprehensive identification of competing systems and providing quantitative criteria for the evaluation of conceptual design schemes. Chen et al. [6] proposed three bionic design methodologies by combining feature transfer with topology. While these studies collectively demonstrate the efficacy of BioTRIZ in guiding bionic design, its inherently cross-domain nature may impede the precise extraction of biological knowledge [30]. Furthermore, the abstract character of the TRIZ inventive principles underscores the critical need for BioTRIZ to improve the objectivity in selecting conflict parameters.

### 2.2. Technological Evolution Theory

The fundamental premise of the technological evolution is to forecast the trajectory of the system in forthcoming research and development activities, thereby informing the advancement of novel products or subsequent product iterations [31,32], as illustrated in Table 2. Investigations and assessments concerning the principles governing technological evolution predominantly rely on expert experience. While the validity of these principles has been extensively corroborated, the inherent subjectivity associated with qualitative analysis remains an area requiring significant refinement [33].

To address the aforementioned challenges, prior research has developed hierarchical frameworks for modeling technological evolution and predictive models of product systems from both macro and micro perspectives. At the macro level, product systems have been conceptualized as couplings of multiple technologies. For instance, Wang et al. [34] employed an automated technology selection method based on generative topographic mapping (GTM) to construct a vector matrix comprising patents and their associated topics, thereby identifying and evaluating technological opportunities. Similarly, Valverde et al. [35] enhanced the retrieval of domain-specific knowledge during technology evolution analysis by leveraging physical phenomena implied or accentuated by functional pivots, thereby improving comprehensiveness. At the micro level, product modeling has been approached as a collaboration of functional networks. Wu et al. [36] mapped cross-domain functional knowledge within patent repositories by calculating functional similarity metrics, effectively supporting the extended design of intelligent service systems for products. Li et al. [37] applied TRIZ functional bases to extract implicit innovation information embedded within patent texts across diverse domains, facilitating designers in transcending knowledge boundaries inherent in iterative product development processes. Bai et al. [8] applied BioTRIZ to address the multi-conflict challenges encountered by products transitioning toward sustainable concepts, thereby augmenting the traditional operation domain with considerations of green design. Existing research particularly highlights the cross-domain characteristics in the evolution of product technologies, underscoring the utility of patents as a dependable source of information for discerning the principles governing technological development. These studies illustrate the potential of technology evolution theory to enhance BioTRIZ and offer valuable insights for developing front-end methodologies in bionic design. Nonetheless, these studies have yet to develop a comprehensive framework for integrating the theory of technological evolution into the biomimetic design process. This gap poses challenges for designers attempting to establish a systematic mapping between technological domain knowledge and product functionality within cyclical constraints, particularly when relying on limited design experience.

## 3. Methodology

### 3.1. Analysis of Technological Evolution Driven by Patents and LLMs

Within the framework of technological evolution theory, the ideal goal represents a tangible manifestation of the evolutionary trajectory of the product system [38]. The development of this ideal goal is grounded in a comprehensive analysis of LTE. To enhance the precision of both the technological evolution assessment and the determination of the ideal goal, this study utilizes a patent information database as the primary data source. This approach facilitates the guidance of LLMs in analyzing the patent evolution trajectory of the design object.

#### 3.1.1. Patent Search and Preprocessing

The information contained within patent documents can be broadly categorized into structured data, which adheres to specific formatting requirements, and unstructured data, presented in natural language, as illustrated in Table 3. Both categories encompass details pertaining to the technological domain of the patent as well as its principal innovations. Accordingly, this study extracts features of technological evolution from patent texts by employing two types of retrieval terms: primary retrieval terms and descriptive retrieval terms. The retrieval field is defined as:

(1) $$\left\{\begin{array}{c} Filing\;time,\;(t_{0},t_{1})\\ Main\_Query,(s_{a},s_{b},\ldots ,s_{X})\\ Des\_Query,(s_{a1},s_{a2},\ldots ,s_{Xn}) \end{array}\right.$$

Here, the main query terms primarily encompass design objects, which are typically product components. Descriptive query terms include the technical systems of the products and their related extensions. Given the potentially large volume of data retrieved, this study analyzes the architecture of the target domain by calculating the degree of membership between IPC codes and the target field. This approach ensures that the patent database retains cross-domain characteristics while extracting descriptive information pertinent to the relevant technical areas. The degree of membership can be calculated by using the following equation:

(2) $$I\left(X;Y\right)=\sum_{x\in X,y\in Y}p\left(x,y\right)\log\left(\frac{p\left(x,y\right)}{p\left(x\right)p\left(y\right)}\right)$$

In Equation (2), x represents the target search field and Y denotes the set comprising the IPC numbers within the database. The term $p\left(x,y\right)$ signifies the joint probability of the simultaneous occurrence of the target field and the IPC number, while $p\left(x\right)$ and $p\left(y\right)$ correspond to the individual probabilities of their respective occurrences. Following this, patents that include fewer than the top k IPC numbers are excluded, and the patent database is accordingly updated.

#### 3.1.2. Patent Feature Extraction

The dimensionality of patent features holds significant importance, serving as a fundamental basis for aligning LTE via the mapping mechanism. Consequently, the patent features developed in this study encompass variations in the quantity or function of components ($P_{c1}$), alterations in the structural configuration ($P_{c2}$), and modifications in the parameters between elements ($P_{c3}$). These features are employed to encapsulate the unstructured information contained within patents, as detailed in Table 4.

It is important to highlight that the IPC serves as a standardized technical categorization framework that accurately reflects the technological domain of a given patent. Consequently, this study employs the assembled descriptive sets $Y^{*}$ corresponding to the top k IPC index, in conjunction with patent features, to develop the corpus D.

(3) $$D=\left\{d_{1},d_{2},\ldots ,d_{k}\right\}$$

To enhance the identification of significant terms within the corpus, this study utilizes the TF-IDF algorithm for analysis. The significance of key terms in this study is defined as follows:

(4) $$\omega_{ij}=\frac{F_{t}}{d}\times \log\frac{N}{N_{t}+1}$$

In Equation (4), $F_{t}$ represents the frequency of the term t within a given document, d signifies the total word count of the document, N indicates the total number of patents included in the patent database, and $N_{t}$ corresponds to the number of documents in which the term t appears.

Subsequently, the latent dirichlet allocation (LDA) model is employed to establish the correspondence between key terms and topical feature dimensions, while Word2Vec is utilized to transform the textual representation of key terms into their respective word vector form. Upon obtaining the word vector representations of the key terms, it becomes possible to retrieve text segments that exhibit the highest similarity to the patent features by analyzing the correlation between the patent text and the anchor points:

(5) $$r=exp\left(\omega_{ij}\cdot {\left\|v_{t}-v_{D}\right\|}^{2}\right)$$

In Equation (5), $\left\|\cdot \right\|$ represents the 2-norm, which is the Euclidean distance between the word vectors of key terms and those of the patent text. During the process of transferring patent feature information to prompt, this study proposes an adaptive method to quantify the significance of the patent feature text relative to the prompt, thereby determining whether it should be incorporated into the prompt framework:

(6) $$S\left(t,C\right)=\exp\left(-\frac{r}{\tau }\right)\cdot \Phi \left(W\right)$$

In Equation (6), τ serves as a control parameter to regulate the degree of dispersion between features and text; Φ is an adaptive function designed to adjust the feature extraction strategy according to the context window size of the selected LLMs. Its purpose is to adaptively push away the text segments that are distantly related to patent features and key terminology, thereby enhancing the precision of prompt generation [39]. In this study, we employ the subject-action-object (SAO) method to standardize information within unstructured patent texts. SAO represents relationships among entities in a process by constructing tuples composed of “subject–action–object,” where the subject and object are typically nouns or pronouns denoting the executor and the recipient of the process, respectively, and the action is generally a verb characterizing the attribute of the process. In complex textual contexts, SAO can be combined with dependency parsing and part-of-speech analysis to further improve the efficiency and accuracy of building feature corpora. Given that these related methodologies are well-established and fall outside the primary focus of this study [40,41], they will not be elaborated upon herein.

#### 3.1.3. Identification of the LTE Driven by LLMs

**Figure 2:**
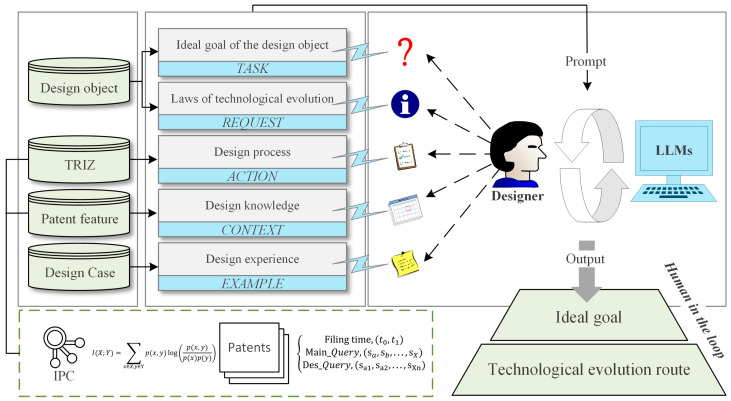
The prompt framework for guiding LLMs to assess LTE.

LLMs pretrained on general-domain data have been extensively employed to address practical engineering challenges [42,43]. To mitigate the risk of “hallucinations” generated by LLMs [44], we have developed a reliable prompt framework based on the TRACE methodology. The TRACE comprises five core components: Task, Request, Action, Context, and Example. This framework enables the integration of patent features alongside the problem-solving processes of TRIZ theory as embedded knowledge and reasoning chains within a constrained prompt window, thereby reducing the cognitive burden on designers when correcting LLMs outputs. For ease of illustration, Figure 2 shows the prompt framework we have constructed.

Specifically, this study explicitly defines the task assigned to the LLMs as conducting an in-depth analysis of the ideal goals of the design target. Within the request module, the LLMs are required to elaborate comprehensively on the LTEs associated with these ideal goals. The construction of these two modules aims to emphasize that the LLMs’ analysis of the product’s ideal goals should be grounded in an examination of the technological evolution pathways, thereby ensuring the completeness and interpretability of this analytical step. The action module guides the LLMs to investigate features such as technical principles, innovations, and application scenarios contained within patent databases, thereby supporting analysis tasks related to technological systems. The context module provides the LLMs with a structured cognitive framework and problem-solving strategies, incorporating relevant concepts and implementation processes from TRIZ. Additionally, this module includes an interface for accessing patent databases, ensuring that the LLMs can effectively retrieve the necessary patent information to facilitate efficient information exchange and utilization. The example module requires the provision of design cases or related texts from the research team. These cases and texts serve as practical exemplars, offering the LLMs essential contextual references for understanding the design scenarios.

### 3.2. Acquisition and Design of Biological Prototypes

In this study, we refer to the parameters that exhibit improvement throughout the evolution of a product system as improvement domains, whereas parameters that impede the optimization process or degrade during the system evolution are classified as deterioration domains. In this study, the idealized design objectives of the product technology system are forecasted by introducing a technological evolution pathway. The resulting ideal goals are then analyzed in comparison with the current state of the product. Based on this, relevant inventive principles are identified by mapping the conflicting parameters onto the PRIZM [25], thereby facilitating the retrieval of biological prototypes from biological databases. Initially, the retrieved biological prototypes undergo a preliminary screening process, as outlined in the methodology proposed by Bai [29], to exclude prototypes that are unreasonable or difficult to interpret. Subsequently, a similarity function is developed to assess the design opportunities δ between biological systems and product technology systems:

(7) $$\delta =\omega_{1}S_{f}+\omega_{2}S_{c}$$

In Equation (7), $\omega_{i}$ denotes the weighting coefficient of the parameter term, the value of which can be established by a domain expert. S represents the similarity between the biological prototype and the designed object, while f and c correspond to the system level and environmental level, respectively. Notably, the system level is further subdivided into five distinct categories: functional, structural, morphological, characteristic, and behavioral layers, as delineated in this study. The allocation of similarity degrees across these categories is presented in Table 5.

A larger value of δ indicates a stronger similarity between the selected biological system and the product system of the design target. Conversely, a smaller value suggests that the chosen biological system holds limited heuristic value for the design object. Therefore, the items within the initial biological prototype database are sequentially ranked to identify key biological prototypes, which serve as the basis for subsequent extraction of biological knowledge.

**Figure 3:**
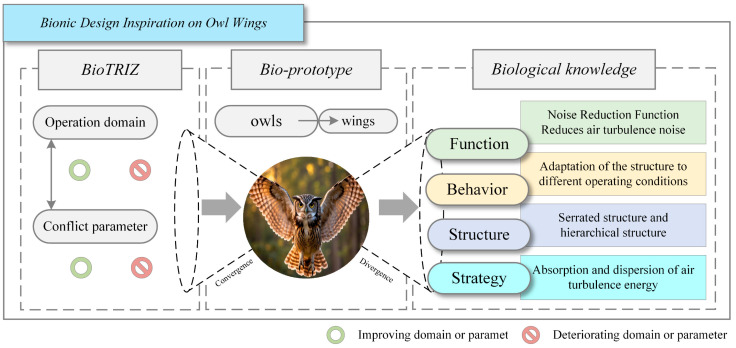
The standardized knowledge model based on BioTRIZ.

To enhance the objectivity and accuracy of biological knowledge representation, this study extracts the knowledge contained within biological prototypes across four hierarchical levels: function, structure, behavior, and strategy. For ease of illustration, Figure 3 illustrates the results of biological knowledge extraction from the owl wing prototype.

The biological knowledge applicable to addressing the engineering problem is examined in the context of the specific challenges and requirements of the design object. Following this analysis, the relevant biological insights are translated into the engineering domain to develop an initial conceptual design solution.

### 3.3. Evaluation of Conceptual Schemes

The MPV is an evaluative model that comprehensively integrates market, product, and value factors [45,46], as defined in follows:

(8) $$MPV=\frac{characteristic\;of\;the\;product}{purchase\;cost\;to\;the\;user}$$

To accurately identify the key characteristics of the product, this study employs data mining methods on online customer reviews to extract user requirements, subsequently constructing a hierarchical framework within the characteristic domain. The comprehensive methodology is described as Algorithm 1.
**Algorithm 1** Pseudocode for the construction of characteristic domain.**Input:** Original Reviews: reviews, Max Topic Candidates: t, Top Words per Topic: k**Output:** Characteristic DomainInitialize preprocessed_reviews**for** reviews **do**      tokens = TOKENIZE(reviews)      filtered_tokens = REMOVE_STOP_WORDS(tokens)      cleaned_tokens = REMOVE_SPECIAL_CHARS(filtered_tokens)      lemmatized_tokens= LEMMATIZE(cleaned_tokens)      preprocessed_reviews.append(lemmatized_tokens)**end for**tfidf_matrix = TF-IDF(preprocessed_reviews)Initialize characteristic_domain**for** num_topics **from** 2 **to** t **do**      lda_model = TRAIN_LDA(tfidf_matrix, num_topics)      coherence = COMPUTE_COHERENCE(lda_model, preprocessed_reviews)      diversity = COMPUTE_DIVERSITY (lda_model, preprocessed_reviews)      current_score = coherence * diversity      **if** current_score > best_score **then**            best_score = current_score            optimal_lda = lda_model      **end if****end for**characteristic_domain = EXTRACT_TOPICS_WITH_TOP_WORD(optimal_lda, k)**Return** characteristic domain

In practical biomimetic design, the presence of multiple improved components leads to an increase in the number of possible final combination schemes. To enhance design efficiency, this study employs orthogonal design combined with Fuzzy-AHP to evaluate the combination schemes. Orthogonal design enables a systematic assessment of the effects of multiple factors on outcomes with a reduced number of experimental trials. In this context, the improved regions are defined as “factors,” and the conceptual design schemes within each region are defined as “levels.” Suppose there are k improvement regions $\left\{D_{1},D_{2},\ldots ,D_{k}\;\right\}$, where the i-th region generates $m_{i}$ conceptual design schemes $\left\{S_{i1},S_{i2},\ldots ,S_{i3}\;\right\}$. However, since the number of schemes across regions is generally inconsistent, a quasi-level method is employed to optimize the constructed orthogonal array $L_{n}\left(m^{k}\right)$. First, the maximum number of levels is determined as $q=max\left(m_{1},m_{2},\ldots ,m_{k}\right)$. For any factor i with $m_{i}\left(m_{i}<q\right)$, $\left(q-m_{i}\right)$ additional levels are supplemented. This process can be modeled by the following equation:

(9) $$\left\{\begin{array}{c} x_{i}^{j}=x_{i}^{\left|\left(j-1\right)\mathrm{mod}m_{i}\right|+1}\\ s.t.\;\;j>m_{i} \end{array}\right.$$

In Equation (9), $x_{i}^{j}$ represents the j-th level of factor i. $m_{i}$ denotes the number of levels for factor i. The mod is used to cyclically repeat the levels of i when $j>m_{i}$. $\left|\left(j-1\right)\mathrm{mod}m_{i}\right|+1$ ensures that the level index wraps around within the range $\left[1,\;m_{i}\right]$, allowing the addition of extra levels by repetition. Upon obtaining the product characteristic domain and the orthogonal array, an expert panel is convened to calculate the weights of the characteristic indicators. Subsequently, a fuzzy complementary judgment matrix $A={\left(a_{ij}\right)}_{n\times n}$ is constructed. This procedure employs a scaling method ranging from 0.1 to 0.9. The equation for calculating the weight vector $W={\left(w_{1},w_{2},\ldots ,w_{n}\right)}^{T}$ using a fuzzy judgment matrix is as follows:

(10) $$w_{i}=\frac{\sum_{j=1}^{n}a_{ij}+\frac{n}{2}-1}{n(n-1)},i=1,2,\ldots ,n$$

In Equation (7), $w_{i}$ denotes the weight value of the i-th feature.

When the fuzzy judgment matrix passes the fuzzy consistency test, the weight vector derived from this matrix is adopted as the weight for each evaluation criterion. Based on the experimental design combinations from the orthogonal array, a scoring table for biomimetic product design schemes is constructed. This table collects the comprehensive scores Q1 from the user perspective on the MPV model for each combination scheme, as well as the comprehensive scores Q2 from biomimetic experts on the biomimetic evaluation indicators for each scheme. These scores are then combined with the weight vector W obtained through the Fuzzy-AHP method to perform a weighted calculation, yielding the overall score Q for each scheme, which is computed by the following equation:

(11) Q=W(Q1+Q2)

Finally, the conceptual design scheme with the highest average score across all factors is selected and combined to produce the final conceptual design solution.

## 4. Model Validation

To assess the efficacy of the proposed method, this section presents a case study. From the perspectives of theoretical frameworks and tools, TRIZ, BioTRIZ, and LLMs have been extensively applied across various domains, including engineering equipment [20], robotics [12], and interactive products [47]. This provides a robust foundation for the cross-context applicability of the proposed method. Accordingly, this study selects a floor scrubber as the subject of the case study, primarily for the following reasons: (1) as a representative mechatronic intelligent system, its core operation depends on the coordinated functioning of multiple components; (2) it must satisfy the adaptive requirements of user scenarios and interactions; and (3) in recent years, this product has accumulated substantial user feedback via e-commerce platforms, highlighting clear areas for improvement. These characteristics closely correspond with the applicable context of the proposed method. During the enhancement process of the intelligent floor scrubber undertaken by the partner enterprise in this study, a survey was conducted to identify the primary components necessitating improvement, namely the roller brush cover, the rotating shaft, and the wastewater tank. For ease of illustration, Table 6 details the specific problems associated with the product.

In addressing these challenges, this section employs the proposed methodology to devise an optimized solution targeting the identified areas for improvement, concurrently validating the efficacy of the approach advanced in this research.

### 4.1. BioTRIZ-Based Design Analysis

#### 4.1.1. Confirmation of Corpus

During the case study, the patent databases consulted included those of the China National Intellectual Property Administration (CNIPA), the United States Patent and Trademark Office (USPTO), and the International Bureau of the World Intellectual Property Organization (WIPO). An initial search yielded a total of 36,617 patent records. Subsequently, through a detailed analysis of the patent texts, patents exhibiting a high degree of relevance were extracted.

**Figure 4:**
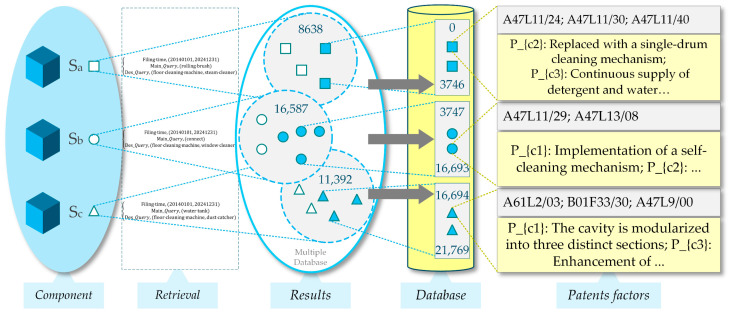
Patent retrieval process utilizing multiple databases.

During the screening process, the technical characteristics of each component’s relevant domain were analyzed according to the method proposed in this study, thereby further excluding patent data with low relevance to the improved components. In this context, the parameter k was set to 20. For ease of presentation, both the results of this process and a portion of the collected corpus D are summarised in Figure 4.

#### 4.1.2. Analysis of LTE and Ideal Goals Based on LLMs

Based on the methodology and procedures proposed in this study, the LTE of three components requiring improvement were identified and subjected to in-depth analysis. Firstly, the ideal goal of $s_{a}$ aims to achieve a self-cleaning function by adhering to L8. This objective seeks to reduce manual intervention and increase the system’s level of automation, enabling the system to autonomously remove stains during operation. However, the current roller brush housing has not yet realized this self-cleaning capability, necessitating the integration of additional functionalities to fulfill this requirement.

The ideal goal for $s_{b}$ follows L9, with the intent to balance operability and protection. By coordinating adjustments between rigidity and flexibility, the rotating shaft can maintain satisfactory control performance while effectively safeguarding internal circuits and water inlet/outlet pipelines. Compared to existing designs, the current rotating shaft struggles to simultaneously meet these demands, thus requiring coordinated optimization of its structural parameters.

The ideal goal for $s_{c}$ is to improve wastewater treatment efficiency and system automation through L8. The existing product employs a multi-layer filtration system to separate wastewater from solid micro-debris. Although this improvement has increased filtration efficiency, it has also introduced greater structural complexity and additional filtration layers, necessitating a balanced trade-off between efficiency and complexity. By clearly defining these goals, the conflict domain for improvement components have been identified, as detailed in Table 7. Here, the Y denotes inspired principle

#### 4.1.3. Identification of Bio-Prototype

In this section, we first obtained biological prototypes from the BioTRIZ biological prototype database based on inventive principles. Ultimately, the inventive principles and their corresponding biological prototypes mapped to the improved components are presented, as shown in Appendix B.

**Figure 5:**
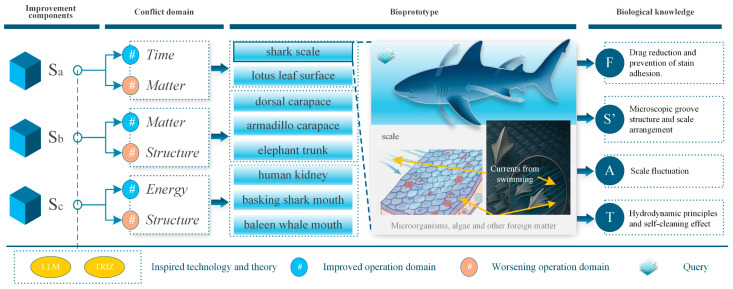
The process of extracting biological prototypes and knowledge.

Upon thorough analysis, $s_{a}$ ultimately designated shark skin scales and lotus leaf surfaces as the principal biomimetic prototypes subsequent to an initial screening process. $s_{b}$ identified the dorsal carapace of woodlice, the elephant trunk, and the armadillo carapace as supplementary biomimetic prototypes. Meanwhile, $s_{c}$ selected the human kidney, the oral cavity of the megamouth shark, and the oral cavity of the baleen whale as further candidate biomimetic prototypes. Comprehensive extraction results are presented in the Appendix A. Here, F denotes function; S’ represents structure; B indicates behavior; and S corresponds to strategy. For clarity, Figure 5 provides an overview of the aforementioned work.

### 4.2. The Development of Evaluation Metrics

The design team established in this study systematically developed six conceptual design schemes based on the aforementioned findings. Detailed schemes are provided in Appendix C. A total of eighteen possible combinations of these conceptual frameworks were identified. This section, together with the following one, utilizes the proposed methodology to ascertain the most effective combination scheme.

**Figure 6:**
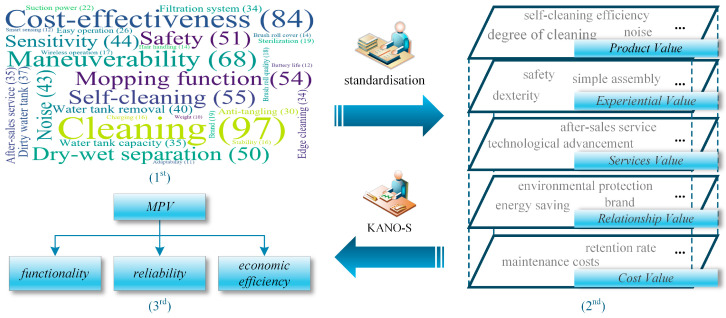
The construction process of the MPV model.

In this section, we conduct an analysis of customer reviews pertaining to best-selling products from both partner companies and competing brands, utilizing data obtained from e-commerce service platform. A total of 4689 online reviews were collected, from which 2647 reviews with ratings of three stars or below were selected. After removing default and invalid entries, 824 valid reviews remained for further analysis. Natural language processing techniques were then employed to preprocess the textual data, followed by tokenization and feature weighting using the TF-IDF algorithm. Through evaluation and ranking, top 30 key terms were extracted. By integrating semantic analysis of them, latent user needs embedded within the reviews were thoroughly examined and mapped onto MPV model, as shown in Figure 6.

Within the MPV model, functionality (#1) refers to the cleaning and filtration capabilities of the floor scrubber during actual operation, reflecting the overall maneuverability and efficiency of the machine. Reliability (#2) denotes the overall durability and safety of the device. Economic efficiency (#3) pertains to the manufacturing and maintenance costs associated with the floor scrubber. Furthermore, in the evaluation criteria for biomimetic design. Functional appropriateness (#4) assesses the degree to which the conceptual design, utilizing biomimetic information, aligns with the engineering problem. Behavioral appropriateness (#5) evaluates the adaptability of the biomimetic actions within the conceptual design to the dynamic characteristics of the product itself. Structural appropriateness (#6) measures the coordination between the structural knowledge derived from biomimetic information and the overall structural configuration of the product.

### 4.3. The Evaluation of Design Schemes

During the case study, an $L_{9}\left(3^{3}\right)$ orthogonal array was constructed based on the design scheme. Due to the misalignment of the levels of the first factor in this orthogonal design experiment, the quasi-level method was employed to improve the experiment by supplementing the missing level in the first factor with design parameter A2. The analysis of the orthogonal design is presented in Table 8.

**Figure 7:**
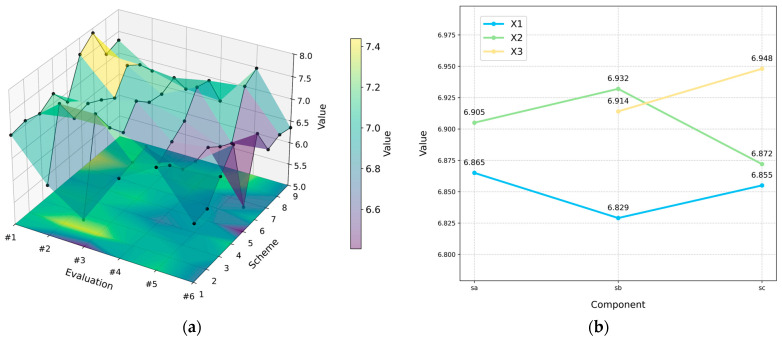
The results of the statistical analysis: (**a**) shows the comprehensive average scores of each combination scheme; (**b**) the results of the orthogonal analysis.

In this study, an evaluation panel consisting of 20 experts with specializations in biomimetic design and industrial design was convened. And a fuzzy judgment matrix was developed using a scale ranging from 0.1 to 0.9 to ascertain the weights of the different indicators. The findings are summarized in Appendix D. The results of the statistical analysis are presented in Figure 7. Therefore, the combination of A2, B2, and C3 constitutes the optimal configuration.

## 5. Discussion and Conclusions

It is noteworthy that existing studies have confirmed that LLMs have attained a level of knowledge comparable to that of global expertise [48]; however, the phenomenon of hallucination remains a critical factor undermining the effectiveness of proposed methods. To address this issue, we conducted experiments to evaluate the efficacy of the proposed approach. We selected Recall@3 and Hits@1 as the primary metrics for assessing the generated results [44]. Recall@3 refers to the proportion of cases in retrieval tasks where at least one relevant result appears within the top three retrieved items, thereby measuring the model’s ability to cover pertinent information. Hits@1 denotes the proportion of instances in which the top-ranked retrieval result is relevant, reflecting the model’s precision in accurately identifying relevant information. Here, we further refined the criterion for relevance in Hits@1 by requiring consistency with human expert judgments, as achieving a high score under this stricter standard is crucial for validating effective collaboration between LLMs and human designers. The experimental results are presented in Table 9.

The results presented in Table 9 demonstrate that our proposed method outperforms both the standard prompting approach and the TRACE prompting method in the Recall@3 evaluation metric. Corresponding improvements are also observed in the Hits@1 metric. These findings indicate that the integration of patent information effectively enhances the accuracy of large language models in determining the LTE. This conclusion further substantiates the assertion made in the literature review section that patents serve as a dependable source of information for design. Moreover, it highlights the potential of large language models to provide human designers with more relevant and trustworthy guidance in biomimetic design.

**Figure 8:**
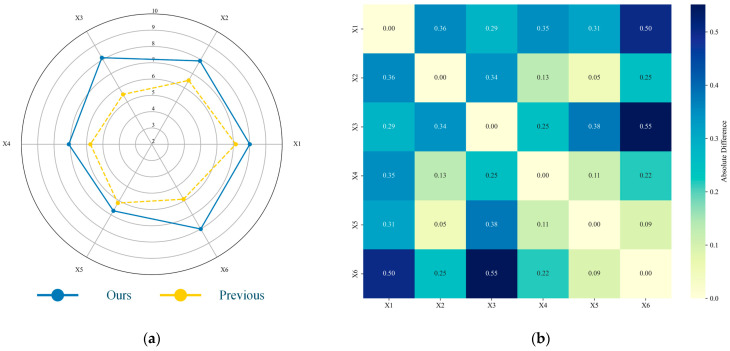
The evaluation and analysis results based on the Delphi method: (**a**) presents the findings derived from the third round of assessment carried out utilizing the Delphi method; (**b**) shows the variations in the correlations among the indicators.

Furthermore, the design team established during the research process, consisting of 31 members who all applied the proposed method in the design phase of this study and possess over two years of experience related to TRIZ, was reconvened to participate in the evaluation of the proposed method. This assessment was conducted based on six core criteria: Effectiveness (#X1), Efficiency (#X2), Learnability (#X3), Robustness (#X4), Repeatability (#X5), and Enlightening (#X6). Utilizing the Delphi method over three rounds with a 10-point rating scale, the team evaluated both the proposed method and previous approaches. The evaluation results are presented in Figure 8.

Based on the evaluation results, the improvements observed across all metrics substantiate the advancement of the proposed method. Specifically, the improvements observed in X1, X3, X4, and X6 suggest the feasibility of the TRIZ-based approach in enhancing the confidence of LLMs during design activities. aligning with the conclusions presented in [33]. Regarding correlation, the proposed method exhibits generally stronger and more positive inter-metric relationships. For instance, it was observed that some participants tend to engage with the visualization of the LLMs’ reasoning processes, which contribute to the increased correlation between X3 and X6.

In the front-end design stages, this study systematically integrates the theory of technological evolution into biomimetic design, effectively reducing the randomness encountered by designers when determining the BioTRIZ operation domain and extracting biological knowledge. During the scheme evaluation stage, an efficient selection of design schemes is achieved through an integrated approach with orthogonal design and Fuzzy-AHP methods. Furthermore, this paper proposes a robust prompt framework to guide LLMs in supporting design activities based on TRIZ methodologies. Specifically, the LLMs analyzes the LTEs of products to predict their ideal goals. This contribution mitigates the reliance of TRIZ-based methods on designer experience and addresses potential subjectivity limitations, while also offering novel insights at the methodological innovation level.

In evaluating this study, we argue that current prompt frameworks are significantly reliant on the completeness and prior structuring of databases. This conclusion is sup-ported by the relatively lower performance observed in indicators X4 and X5 compared to other metrics. Subsequent research will implement class-incremental techniques [39] that support the gradual addition of new data alongside design progression. This approach will facilitate knowledge transfer on a small scale during the initial phases and progressively broaden the data range, thereby enhancing the method’s adaptability to design scenarios characterized by limited data availability. Furthermore, due to constraints related to funding and time duration, the biological data employed in this study were sourced from external databases. We will involve the experimental assessment of the impact that various biological databases have on the reliability of outcomes. Additionally, we are committed to creating an open-source biological knowledge database designed to facilitate biomimetic design.

## Figures and Tables

**Table 1:** The PRIZM of BioTRIZ.

Operation Domains	Matter (M)	Structure (St)	Space (Sp)	Time (T)	Energy (E)	Information (I)
M	13, 15, 17, 20, 31, 40	1-3, 15, 24, 26	1, 5, 13, 15, 31	15, 19, 27, 29, 30	3, 6, 9, 25, 31, 35	3, 25, 26
St	1, 10, 15, 19	1, 15, 19, 24, 34	10	1, 2, 4	1, 2, 4	1, 3, 4, 15, 19, 24, 25, 35
Sp	3, 14, 15, 25	2-5, 10, 15, 19	4, 5, 36, 14, 17	1, 19, 29	1, 3, 4, 15, 19	3, 15, 21, 24
T	1, 3, 15, 20, 25, 38	1-4, 6, 15, 17, 19	1-4, 7, 38	2, 3, 11, 20, 26	3, 9, 15, 20, 22, 25	1-3, 10, 19, 23
E	1, 3, 13, 14, 17, 25, 31	1, 3, 5, 6, 25, 35, 36, 40	1, 3, 4, 15, 25	3, 10, 23, 25, 35	3, 5, 9, 22, 25, 32, 37	1, 3, 4, 15, 16, 25
I	1, 6, 22	1, 3, 6, 18, 22, 24, 32, 34, 40	3, 20, 22, 25, 33	2, 3, 9, 17, 22	1, 3, 6, 22, 32	3, 10, 16, 23, 25

**Table 2:** The laws of technological evolution in modern TRIZ theory.

Code	LTE
L1	Elevated degree of idealization
L2	The uneven development of subsystems
L3	Dynamic growth
L4	Evolution toward a supersystem
L5	Evolution toward microsystems
L6	Integrity
L7	Reduce the length of the energy flow
L8	Enhance controllability
L9	Enhance harmony

**Table 3:** Information and categories contained within a patent document.

Category	Element
Structured data	Patent number, filing date, international patent classification (IPC), etc.
Unstructured data	Specification, claims, abstract, etc.

**Table 4:** Dimensions of patent features.

Index	Features Description
$P_{c1}$	This dimension describes the addition and reduction of components in a technical system, or changes in their functions, and is used to reflect adjustments in system complexity and functionality.
$P_{c2}$	This dimension describes changes in the topological structure of a technical system and the spatial relationships between components, and is used to reflect innovations in system architecture.
$P_{c3}$	This dimension describes the optimization and adjustment of parameters in a technical system, and is used to reflect the optimization of system performance.

**Table 5:** The similarity measures and their corresponding degrees.

Value	Value Standard
0.2	Basically
0.4	Slightly
0.6	Obviously
0.8	Very
1	Extremely

**Table 6:** The improvement component of floor scrubbers.

Code	Component	Issues for Improvement
$s_{a}$	roller brush cover	During operation, the roller brush rotates at high velocity, causing contaminants to accumulate on the external surface of the roller brush housing. This buildup causes unpleasant odors and requires frequent disassembly for cleaning.
$s_{b}$	rotating shaft	To safeguard the internal wiring and the water inlet and outlet pipes, this component is constructed with a rigid framework; however, this design is incompatible with the flexible rotation demanded during use, resulting in reduced maneuverability of the floor scrubber
$s_{c}$	wastewater tank	Particulate matter of various sizes readily clogs the filter screen within the wastewater tank, impeding the efficient separation of wet and dry debris, thereby requiring regular disassembly and rinsing maintenance.

**Table 7:** Mapping results among improvement components, conflict domains, and principles.

Component	Conflict Domain	Invention Principle
$s_{a}$	Time/matter	1(Y), 3, 15(Y), 20, 25(Y), 38
$s_{b}$	Matter/structure	1(Y), 2(Y), 3(Y), 15(Y), 24, 26
$s_{c}$	Energy/Structure	1(Y), 3(Y), 5, 6, 25(Y), 35, 36, 40

**Table 8:** The orthogonal analysis of the design schemes.

Code	$s_{a}$	$s_{b}$	$s_{c}$
1	A1	B1	C1
2	A1	B2	C3
3	A1	B3	C2
4	A2	B1	C3
5	A2	B2	C2
6	A2	B3	C1
7	A2	B1	C2
8	A2	B2	C1
9	A2	B3	C3

**Table 9:** Experimental results on the LTE set.

Basic LLMs	Method	Recall@3	Hits@1
Deepseek-R1	Standard prompt	41.56%	33.76%
	TRACE prompt	67.53%	51.95%
	Ours	77.92%	71.43%
GPT-4o-mini	Standard prompt	38.71%	29.03%
	TRACE prompt	48.39%	45.16%
	Ours	74.19%	70.96%
Claude-3.7-sonnet	Standard prompt	30.19%	28.30%
	TRACE prompt	49.06%	47.17%
	Ours	77.36%	62.26%

## Data Availability

The original contributions presented in this study are included in the article. Further inquiries can be directed to the corresponding author.

## Appendix A

**Table A1:** The comprehensive extraction results of the floor scrubber.

Component	Code	Bio-Prototype	Standardized Knowledge
$s_{a}$	A1	shark skin scale	F: drag reduction and stain prevention; S’: microscopic groove patterns and scale arrangements; B: scale undulation; S: fluid dynamics principles and self-cleaning mechanisms.
	A2	lotus leaf surface	F: self-cleaning and waterproofing; S’: microscopic papillae and nano-scale wax layers; A: hydrophobicity; S: hydrophobicity principles and self-cleaning effects.
$s_{b}$	B1	dorsal carapace of woodlice	F: protection and rotational capability; S’: hierarchical bony plates with flexible connections; B: curling motion; S: integration of rigidity and flexibility and zonal optimization principles.
	B2	armadillo carapace	F: protection and support for locomotion; S’: layered hierarchical architectures; B: combination of rigid and flexible movements; S: strategies to avoid stress concentration.
	B3	elephant trunk	F: flexible operation and protection; S’: muscular layers and connective tissues; B: multi-degree-of-freedom and adaptive movements; S: muscle coordination principles and layered structural optimization.
$s_{c}$	C1	human kidney	F: efficient filtration and waste separation; S’: graded filtration units; B: dynamic regulation; S: modular design and intelligent control systems.
	C2	oral cavity of the megamouth shark	F: efficient filtration; S’: gill raker formations and streamlined morphology; B: water flow passage; S: graded filtration principles and streamlined optimization.
	C3	oral cavity of the baleen whale	F: efficient filtration and protection; S’: baleen formations with layered structures; B: filtration capability; S: graded filtration principles.

## Appendix B

**Table A2:** The biological prototypes identified based on the invention principle.

Component	Invention Principle	Bio-Prototype
$s_{a}$	1	Insect segmentation, gecko tail autotomy, the phenomenon of lotus root fibers remaining connected despite breakage
	15	Visual processing systems, shark skin scales, nest construction behaviors of rock ants.
	25	The surface properties of lotus leaves, human arterial structures, dolphin skin characteristics, bat echolocation mechanisms
$s_{b}$	1	The dorsal exoskeleton of woodlice, the scaled feet of snails, the backs of desert scorpions, the carapace of armadillos
	2	Bioluminescence in fireflies, jet propulsion in jellyfish, the visual system of purple sea urchins
	3	Cicada molting processes, visual processing systems, shark skin scales
	15	The trunk of elephants, arthropod morphology, predatory behaviors of frogs
$s_{c}$	1	Gecko tail autotomy, the oral cavity of the frilled shark, the oral structures of baleen whales
	3	Dragonfly wings, cactus spines, human renal anatomy
	25	The surface characteristics of lotus leaves, dolphin skin, seeds of the maple tree

## Appendix C

**Table A3:** The conceptual schemes developed for intelligent floor scrubber (A).

Code	Conceptual Schemes	Description
A1		Implement microscopic groove patterns inspired by shark skin scales to develop an anti-fouling coating for the roller brush casing. The rapid rotation of the roller brush induces airflow that facilitates the removal of debris, thereby enabling a self-cleaning mechanism.
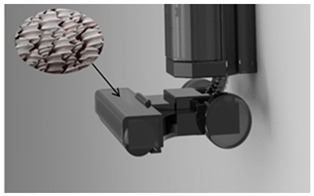
A2		Incorporate the waxy surface architecture derived from lotus leaves to fabricate an anti-fouling layer on the roller brush casing. The superhydrophobic characteristics and material properties of this structure contribute to an effective self-cleaning function.
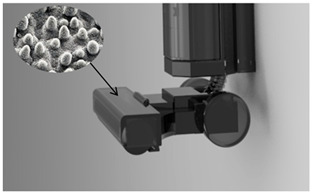

**Table A4:** The conceptual schemes developed for intelligent floor scrubber (B).

Code	Conceptual Schemes	Description
B1		Utilize the hierarchical bony plate configuration of the woodlouse’s dorsal armor to engineer the rotating shaft, integrating both rigidity and flexibility to ensure free rotation while safeguarding internal components.
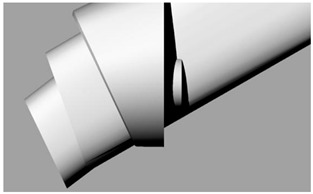
B2		Apply the structural zoning concept observed in the armadillo’s dorsal armor by employing a layered wrapping design that permits multidimensional expansion and contraction, simultaneously providing protection to internal elements.
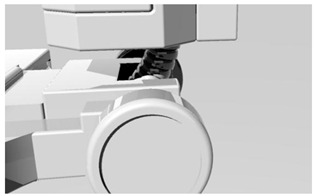
B3		Draw upon the boneless, layered tissue composition of the elephant’s trunk to fulfill the flexibility requirements of the rotating shaft, leveraging the interconnected nature of these tissues to achieve multi-degree-of-freedom movement.
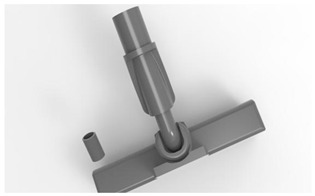

**Table A5:** The conceptual schemes developed for intelligent floor scrubber (C).

Code	Conceptual Schemes	Description
C1		Adopt the modular architecture of the human kidney to establish a hierarchical filtration system, wherein the lower section targets the removal of larger debris and the upper section addresses finer particulates, with dynamic adjustments responsive to the wastewater tank’s condition.
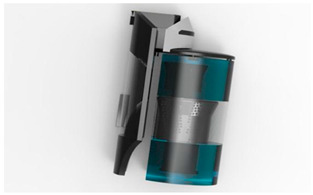
C2		Employ the gill raker structures characteristic of the basking shark’s oral cavity, combined with streamlined design principles, to develop a filtration system that operates from the interior outward, incorporating groove features to mitigate clogging.
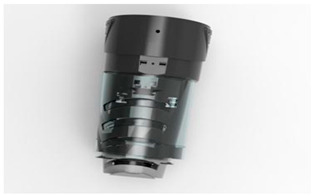
C3		Utilize the baleen structures found in baleen whales to design a bottom-layer filtration system, implementing layered filtration to effectively segregate particulate matter of varying sizes prior to subsequent upper-level filtration, thereby preventing blockages.
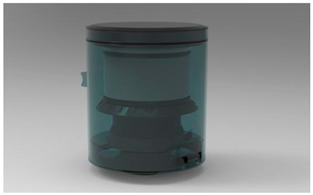

## Appendix D

**Table A6:** The summary of the weights assigned to indicators as evaluated by experts.

Experts	#1	#2	#3	#4	#5	#6
1	0.200	0.157	0.140	0.173	0.163	0.167
2	0.193	0.160	0.140	0.173	0.163	0.171
3	0.177	0.170	0.133	0.177	0.160	0.183
4	0.167	0.160	0.133	0.183	0.177	0.180
5	0.183	0.170	0.127	0.177	0.160	0.183
6	0.187	0.173	0.127	0.173	0.167	0.173
7	0.177	0.160	0.183	0.177	0.170	0.133
8	0.197	0.170	0.127	0.170	0.163	0.173
9	0.200	0.147	0.167	0.162	0.157	0.167
10	0.173	0.171	0.163	0.193	0.160	0.140
11	0.183	0.160	0.140	0.180	0.167	0.170
12	0.170	0.163	0.173	0.197	0.127	0.170
13	0.187	0.183	0.127	0.170	0.163	0.170
14	0.177	0.170	0.176	0.183	0.157	0.137
15	0.197	0.162	0.127	0.170	0.167	0.177
16	0.187	0.147	0.167	0.170	0.162	0.167
17	0.177	0.170	0.133	0.177	0.160	0.183
18	0.192	0.157	0.127	0.177	0.170	0.177
19	0.183	0.160	0.140	0.180	0.167	0.170
20	0.177	0.163	0.133	0.177	0.167	0.183
Weighting factor	0.184	0.164	0.144	0.177	0.162	0.169
